# The Impact of Minimum Invasive Access Cavity Design on the Quality of Instrumentation of Root Canals of Maxillary Molars Using Cone-Beam Computed Tomography: An in Vitro Study

**DOI:** 10.7759/cureus.67705

**Published:** 2024-08-25

**Authors:** Fahad H Baabdullah, Samia M Elsherief, Rayan A Hawsawi, Hetaf S Redwan

**Affiliations:** 1 Basic and Clinical Oral Sciences, Faculty of Dental Medicine, Umm Al-Qura University, Makkah, SAU; 2 Restorative Dentistry, Faculty of Dental Medicine, Umm Al-Qura University, Makkah, SAU; 3 Endodontics, Cairo University, Cairo, EGY

**Keywords:** reciprocating single-file canal transportation, centering ability, cone-beam computed tomography (cbct), traditional access cavity, conservative access cavity

## Abstract

Aim

Minimally invasive dentistry has been facilitated by advances in instruments and restorative materials. This study aims to compare the change in the shaping ability of the RECIPROC blue rotary system in both traditional and conservative access cavities, using cone beam computed tomography (CBCT).

Material and methods

Sixty root canals of 20 artificial maxillary molars were assigned into two groups (n=30 root canals) according to the access cavity design used: Group I: traditional access cavity (TAC) and Group II: conservative access cavity (CAC). CBCT scans of samples were made before and after root canal preparation using the RECIPROC blue rotary system. The shaping parameters are evaluated in root canal transportation and the centering ability. Three CBCT sections per tooth were analyzed at 3, 6, and 9 mm from the apex to assess the canal transportation and centering ability at three levels, apical, middle, and coronal thirds. Data were analyzed using the GraphPad Prism (GraphPad Software, San Diego, CA).

Results

The results of this study showed a significant difference in transportation within the coronal and middle thirds. However, in apical thirds, there were no significant differences. Both groups observed a significant difference in the centering ability in the coronal third.

Conclusion

Within the limitations of this study, CAC can be recommended with caution as an alternative access to TAC.

## Introduction

The main objectives of endodontic therapy are to eliminate or decrease bacteria and their toxins from the pulp and the periapical tissue [1]. Adequate access cavity preparation is essential for detecting, exploring, and properly delivering therapeutic chemo-mechanical cleaning into the root canal system, followed by appropriate 3-D obturation [2]. Therefore, the access cavity could be considered the most essential step in root canal procedures [3].

This objective can be achieved through an appropriate knowledge of root canal morphology using recent technologies, such as cone beam computed tomography (CBCT), magnification, illumination, ultrasonic tips, modified NiTi instruments, and recent filling techniques [4-6].

Minimally invasive concepts, currently advocated as less invasive alternatives to conventional procedures, have a significant impact on endodontic therapy procedures, and these concepts allow for minimally invasive approaches, such as contracted endodontic access cavities for preserving coronal and radicular tooth structure. Consequently, the application of traditional access cavity preparations can be considered a questionable issue as being an invasive procedure [2,7,8].

Root canal treatment failure due to tooth fracture is commonly reported and can be attributed to the significant loss of tooth structure as a result of caries and specific cavity design of endodontically treated teeth [9-11].

Although there is strong support for minimally invasive access cavities, a scientific-based rationale is essential to prove the efficacy of the results of this technique and, eventually, ensure an adequate clinical outcome [12].

Straight line access (SLA) can decrease the chance of iatrogenic errors such as zips, elbows, perforations, and ledges caused by inflexible stainless-steel files that may lead to straightening the curved canals. Although NiTi instruments are more flexible, inadequate SLA might lead to file distortion and cause separation due to cyclic fatigue [13]. These instruments should, ideally, reach the apical foramen. Excessive instrument stress can lead to uncontrolled authority on the instruments that might cause an undesirable issue in the form of procedural errors such as unprepared areas of the canal, ledging, transportation, or zipping [14].

The reciprocating file motion was initially reported as an extension idea of Roane’s balanced force technique [13]. It has many advantages compared to continuous rotation motion, including decreased instrument fatigue and good compliance with canal anatomy with a decreased incidence of procedural errors [15-17].

The RECIPROC system (VDW, Munich, Germany) is constructed of a NiTi alloy named M-wire, created by a thermal treatment technique [18]. This M-wire alloy, combined with the reciprocating motion, increases flexibility and enhances resistance to cyclic fatigue [19]. More recently, RECIPROC Blue emerged with the same design. However, the flexibility of RECIPROC Blue is greater than RECIPROC due to differences in the heat treatment process [18].

The aim of this in-vitro study was to compare the change in the shaping ability of the RECIPROC Blue rotary system in both traditional and conservative access cavities, using cone beam computed tomography (CBCT).

## Materials and methods

Sample preparation

Sixty root canals TrueTooth® replicas of upper molars were used in the study [20]. The samples were scanned using CBCT. The teeth were divided into two groups (n=30) according to the access cavity being tested: conservative access cavity (CAC) and traditional access cavity (TAC). The access cavity was done with the aid of a surgical microscope. A glide path enlargement was done with manual files up to size 20 to reach the working length (WL). A single file in reciprocating motion was used pecking motion about 3 mm with slight apical pressure following the pre-programmed settings of a VDW. Silver RECIPROC motor (VDW, München, Germany), an endodontic engine at the suggested setting (300 rpm on display, 5 Ncm). Patency was maintained using a size 10 K-file between each file. The motion was repeated til the WL was achieved with RB25.

Irrigation was performed between each file using 3 mL of 5.25% NaOCl. After root canal instrumentation was finished 1 mL of 15% ethylenediaminetetraacetic acid (EDTA; Wizard, Rehber Kimya San, Istanbul, Turkey) was used for one minute, and the canals were finally irrigated with 3 mL of NaOCl as a final flush.

CBCT scanning

A CBCT machine (ICAT 17-19, Imaging Science International, Hatfield, PA) was used to scan the samples. The CBCT scans were conducted with the following acquisition parameters: 120 kV, 5 mA, 7 ms of exposure time, voxel size of 0.2-0.25 mm, and a slice thickness ranging from 0.20 to 0.25 mm. Subsequently, each scan was transferred to the digital imaging and communications in medicine (DICOM) format. The DICOM files underwent processing by applying the ImplaStation software (ProDigiDent, IL; www.implastation.com).

**Figure 1:**
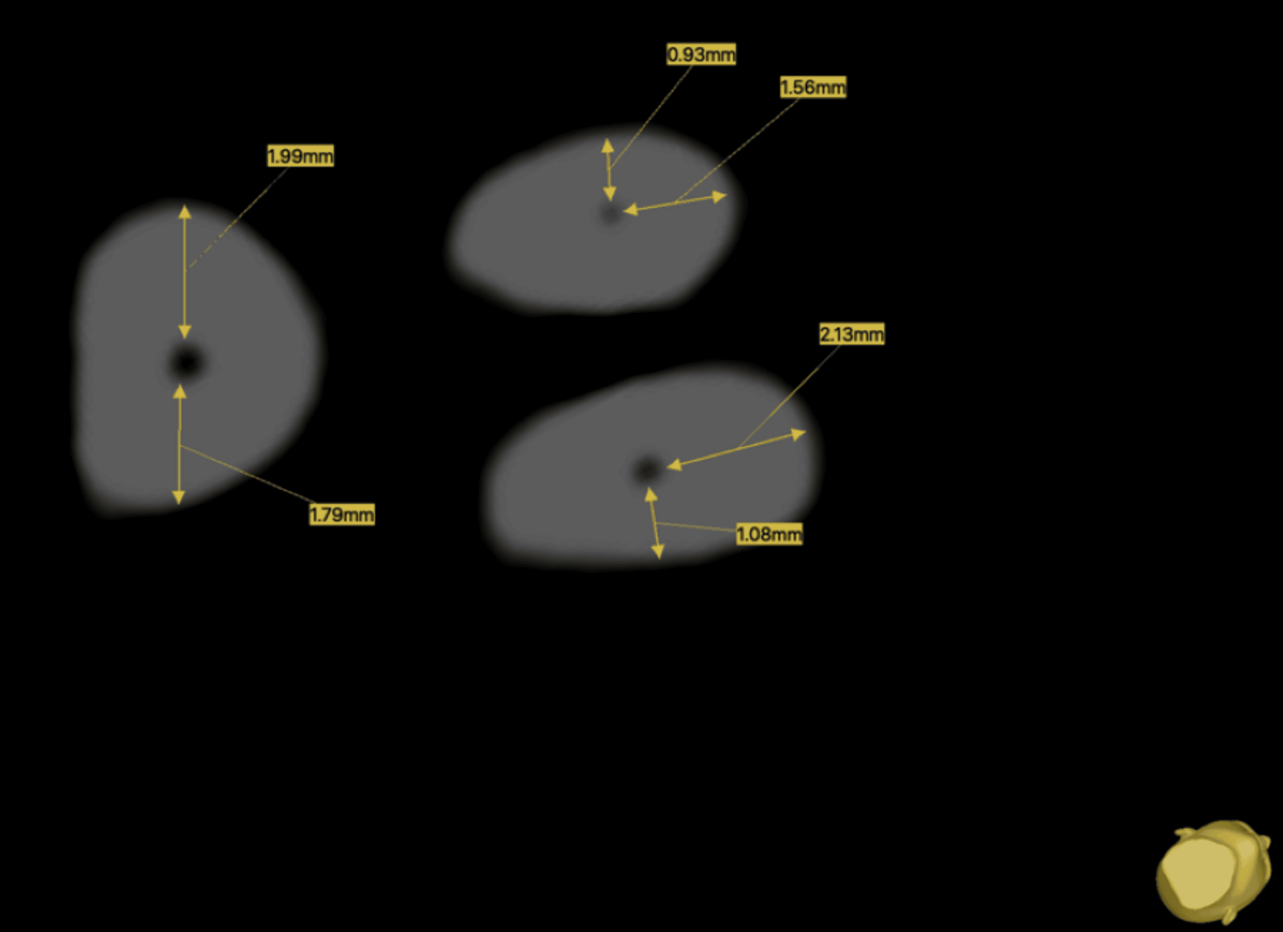
Cross-sectional image at 6 mm from the apex showing representative measurements for each root.

The following methodology was employed to obtain a proper view of the samples in all three anatomical planes (sagittal, coronal, and axial). The longitudinal axis of each sample was oriented vertically on both the coronal and sagittal planes. Three measurement levels at 3, 6, and 9 mm from the apex were demarcated to correspond to the central region of the apical, middle, and coronal thirds of the roots. Measurements were conducted on the axial plane from the outer surface of the root to the outer line of the pulp canal. The representative measurements are shown in Figure 1.

For the mesiobuccal root (D1), measurements were made on the mesial (m) and buccal (b) sides. The amount of transportation was calculated using the formula: $(m1 &minus; m2) &minus; (b1 &minus; b2)$.

The following formula was used to calculate the centering ability: $(d1 &minus; d2) / (b1 &minus; b2)$.

For the distobuccal root (D2), measurements were made on the distal (d) and buccal (b) sides. The amount of transportation was calculated using the formula: $(d1&minus;d2) - (b1-b2)$.

The following formula was used to calculate the centering ability: $(m1 - m2) / (b1 - b2)$.

For the palatal root (D3), measurements were made on the mesial (m) and distal (d) sides. The amount of transportation was calculated using the formula: $(m1 - m2) - (d1 - d2)$.

The following formula was applied for the calculation of centering ability: $(m1 - m2) / (d1 - d2)$.

Here, m1 is the distance from the mesial position of the non-instrumented canal to the outer wall; m2 is the distance from the mesial position of the instrumented canal to the outer wall; d1 is the distance from the distal position of the non-instrumented canal to the outer wall; d2 is the distance from the distal position of the instrumented canal to the outer wall; b1 is the distance from the buccal position of the non-instrumented canal to the outer wall; and b2 is the distance from the buccal position of the instrumented canal to the outer wall.

Statistical analysis

Data were analyzed using GraphPad Prism version 10.0.0 for Mac OS X (GraphPad Software, Boston, MA; www.graphpad.com). The results were statistically evaluated using a full-factorial ANOVA with Tukey’s honestly significant difference (a=0.05). An independent t-test was used to compare the transportation measurements between the traditional and the conservative methods, and the level of significance was set at p<0.05.

## Results

In this study, no instrument fractures, perforations, or ledges happened during root canal preparation. There was a significant difference in canal transportation measurements between the traditional and conservative endodontic access cavities in the coronal and middle thirds (p<0.05).

**Figure 2:**
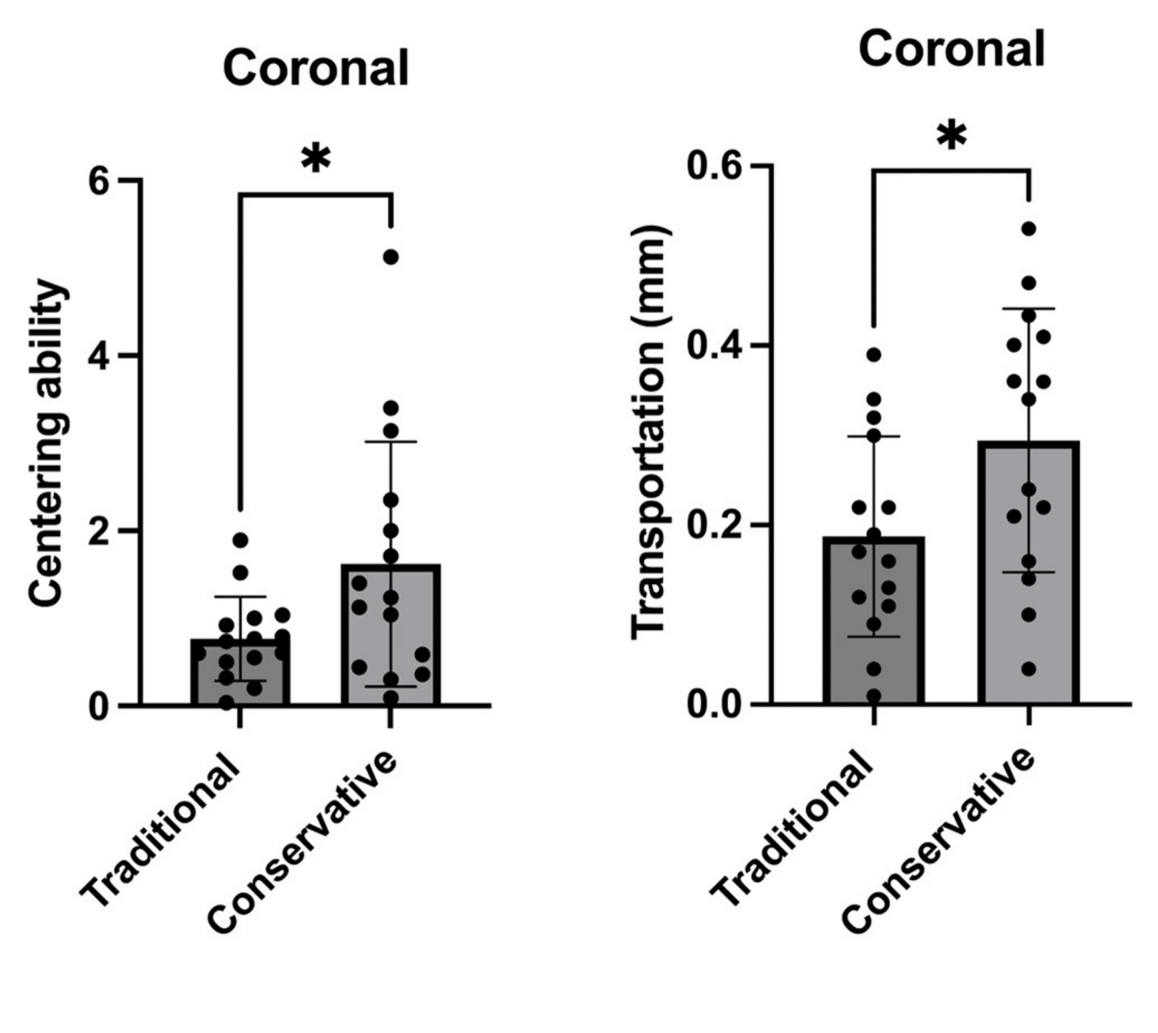
Comparison between the traditional and conservative endodontic access based on centering ability and transportation at the coronal level of all tested roots (n=30). The graphs shows that there was a significant difference between the traditional and conservative endodontic access regarding centering ability and transportation. *p<0.05 was accepted as a significance level.

**Figure 3:**
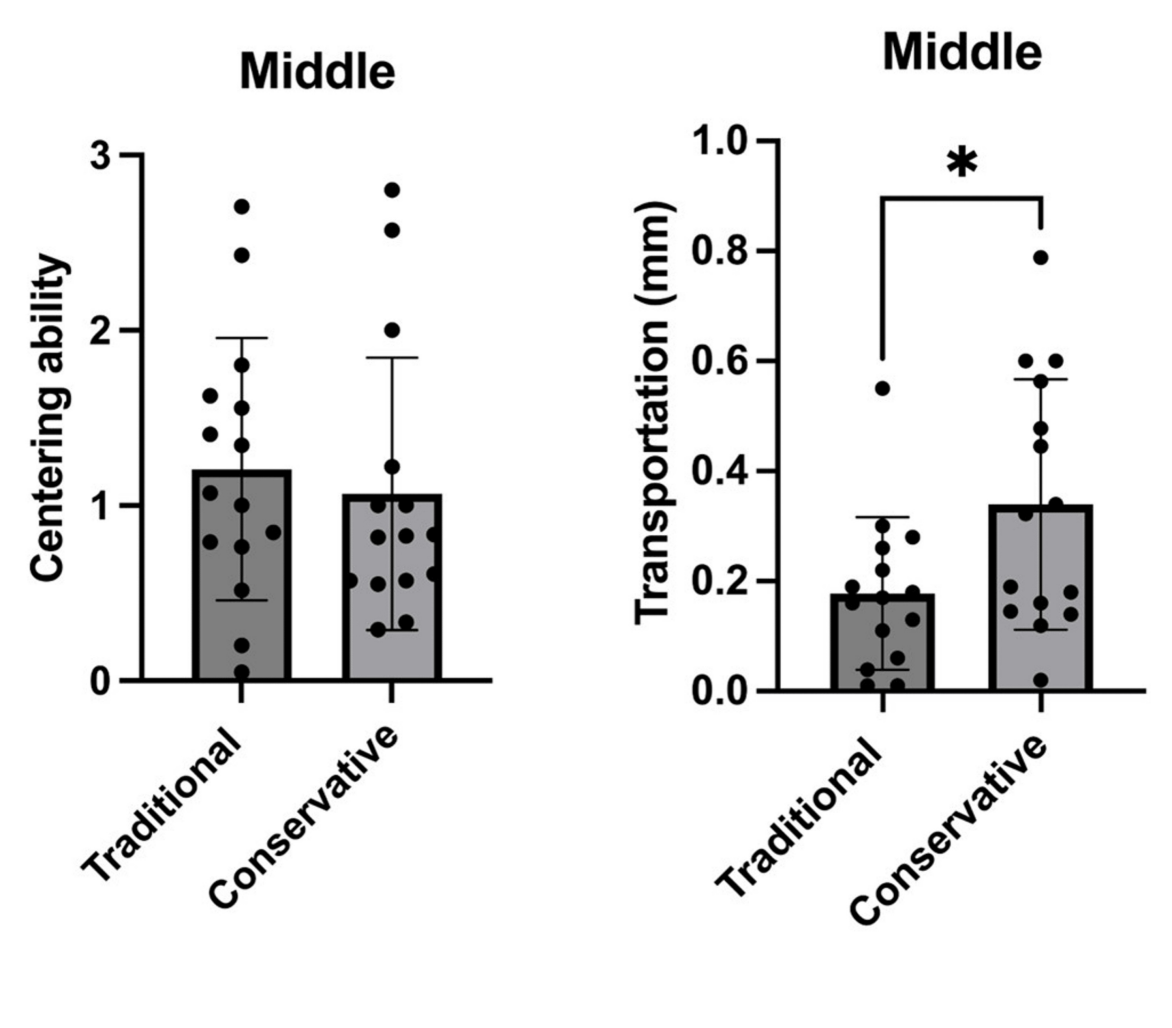
Comparison between the traditional and conservative endodontic access based on centering ability and transportation at the middle level of all tested roots (n=30). The graphs shows that there was a significant difference between the traditional and conservative endodontic access regarding transportation only. *p<0.05 was accepted as a significance level.

**Figure 4:**
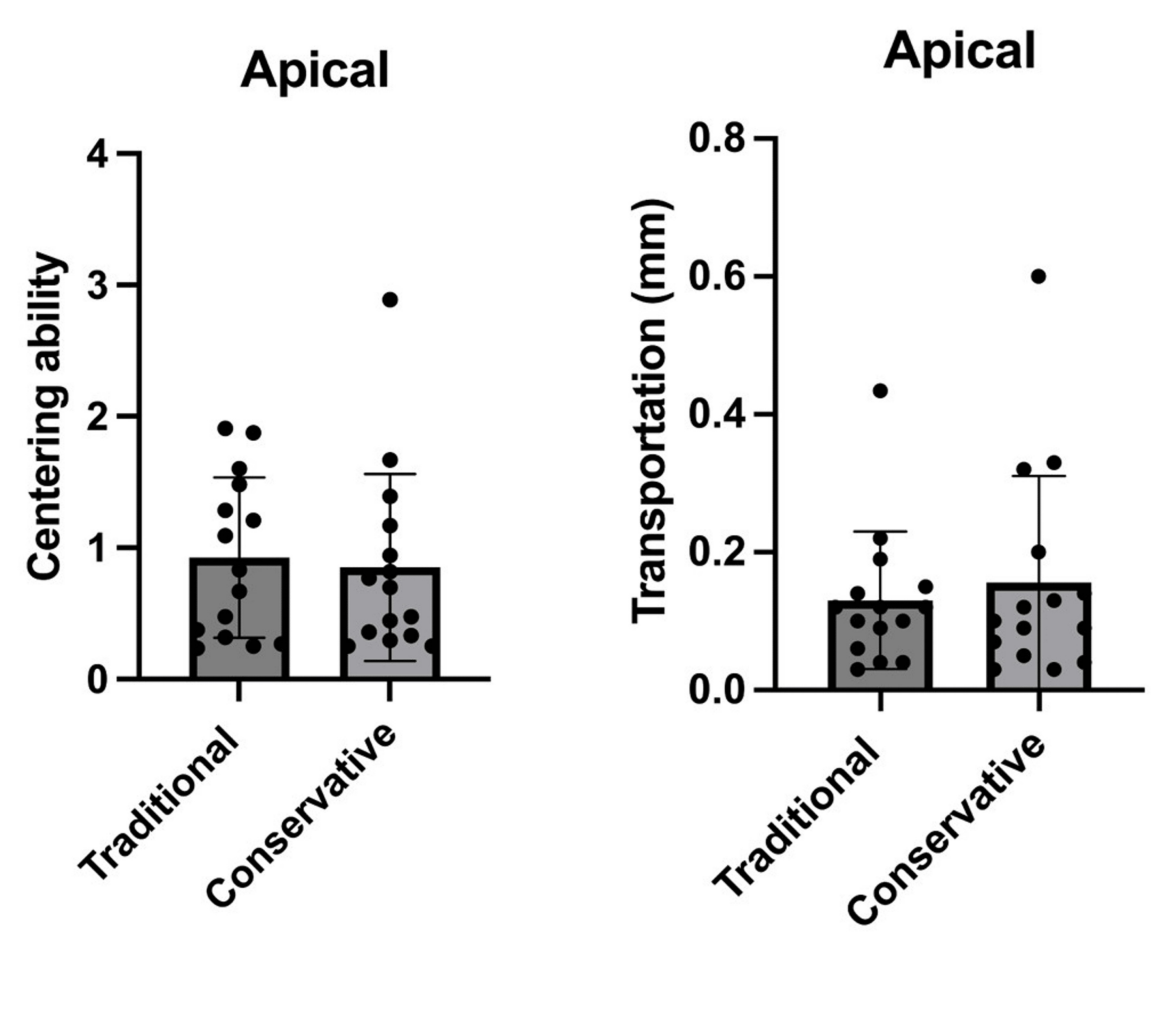
Comparison between the traditional and conservative endodontic access based on centering ability and transportation at the apical level of all tested (n=30). The graphs shows that there was not a significant difference between the traditional and conservative endodontic access regarding centering ability and transportation. *p<0.05 was accepted as a significance level.

Moreover, there was a significant difference at the coronal thirds (p<0.05) in regard to the centering ability measurements in the conservative access cavity groups, as shown in Figures 2-4.

**Figure 5:**
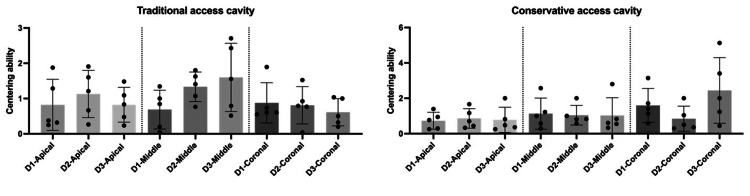
Comparison between the traditional and conservative endodontic access based on centering ability at apical, middle, and coronal levels of all tested roots (D1-D3). The graphs show that there was not a significant difference between all tested roots (D1-D3) for both access cavity designs (traditional and conservative). *p<0.05 was accepted as a significance level.

**Figure 6:**
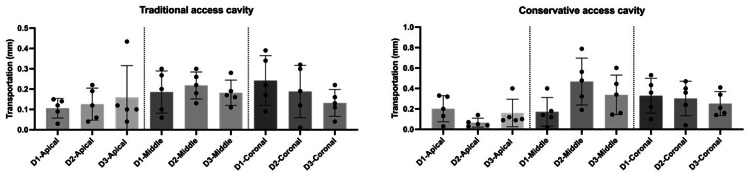
Comparison between the traditional and conservative endodontic access based on transportation at apical, middle, and coronal levels of all tested roots (D1-D3). The graphs show that there was not a significant difference between all tested roots (D1-D3) for both access cavity designs (traditional and conservative). *p<0.05 was accepted as a significance level.

The centering ability and transportation measurements for both traditional and conservative endodontic access designs at the coronal, middle, and apical levels of all tested roots (D1-D3) are presented in Tables 1-3. Figures 5 and 6 show that there was not a significant difference between the tested roots (D1-D3) for both access cavity designs regarding centering ability and transportation values.

**Table 1:** The centering ability and transportation values of the traditional and conservative techniques at the coronal level of all tested roots (n=30). *p<0.05 was accepted as a significance level.

Root	Centering ability		Transportation	
	Traditional	Conservative	Traditional	Conservative
D1	0.607	1.400	0.170	0.433
	0.736	1.125	0.390	0.360
	0.600	0.588	0.090	0.100
	0.550	3.143	0.220	0.220
	1.893	1.714	0.340	0.530
D2	0.792	1.043	0.190	0.240
	0.923	2.350	0.120	0.470
	0.038	0.091	0.010	0.360
	0.769	0.300	0.300	0.400
	1.143	0.364	0.320	0.040
D3	0.792	3.400	0.110	0.340
	0.923	0.444	0.220	0.160
	0.038	2.000	0.040	0.140
	0.769	5.125	0.130	0.410
	1.143	1.235	0.160	0.210
Mean	0.781	1.622	0.187	0.294
SD	0.444	1.397	0.112	0.147
P value	0.033		0.033	

**Table 2:** The centering ability and transportation values of the traditional and conservative techniques at the middle level of all tested roots (n=30). *p<0.05 was accepted as a significance level.

Root	Centering ability		Transportation	
	Traditional	Conservative	Traditional	Conservative
D1	0.200	0.292	0.010	0.020
	0.848	1.000	0.010	0.140
	1.000	1.222	0.060	0.119
	1.345	2.571	0.039	0.180
	0.846	0.971	0.550	0.601
D2	1.625	1.745	0.260	0.478
	2.800	0.818	0.180	0.322
	0.765	0.826	0.130	0.190
	1.071	0.571	0.300	0.788
	1.405	1.000	0.220	0.563
D3	0.792	1.340	0.170	0.160
	0.923	0.444	0.280	0.340
	1.538	1.630	0.190	0.145
	1.769	1.125	0.160	0.445
	1.143	1.035	0.110	0.601
Mean	1.205	1.106	0.178	0.339
SD	0.597	0.566	0.139	0.228
P value	0.619		0.026	

**Table 3:** The centering ability and transportation values of the traditional and conservative techniques at the apical level of all tested roots (n=30). *p<0.05 was accepted as a significance level.

Root	Centering ability		Transportation	
	Traditional	Conservative	Traditional	Conservative
D1	0.250	0.769	0.030	0.200
	1.286	0.943	0.090	0.330
	1.875	0.250	0.150	0.130
	0.318	0.294	0.140	0.030
	0.375	1.391	0.120	0.320
D2	1.600	0.444	0.120	0.040
	0.667	1.167	0.060	0.140
	0.267	1.667	0.040	0.050
	1.909	0.700	0.220	0.070
	1.208	0.333	0.190	0.030
D3	0.235	0.476	0.040	0.100
	0.476	0.250	0.100	0.120
	0.833	0.818	0.100	0.090
	1.481	2.889	0.434	0.601
	1.091	0.360	0.120	0.090
Mean	0.925	0.850	0.130	0.156
SD	0.609	0.712	0.100	0.155
P value	0.760		0.592	

## Discussion

Minimal access cavity preparation and its merits have been issues of much debate during the past few years. For many decades, traditional access has been emphasized to get better visualization and straight-line access for the mechanical instruments to reach the apical third of the root canal. This concept has been reshaped with the introduction of new advanced technology. Access utilizing high magnification prevents the unnecessary removal of cervical tooth structure during endodontic procedures; moreover, the superelasticity of NiTi alloys does not depend on straight-line access to prepare curved root canals [3,4].

CBCT imaging was applied as it provides an accurate, reproducible, three-dimensional estimation of dentin thickness and root canal volume pre and post preparation without damaging the samples [21,22].

Comparing the shaping ability is usually conducted using extracted human teeth or resin blocks. Natural teeth exhibit a high degree of variability in the shape and size of the root canal system [23]. Moreover, it is impossible to standardize anatomical irregularities, which could affect the results. Additionally, the use of natural teeth comes with several drawbacks, including the difficulty of collection, ethical issues, potential for cross-infection, storage challenges, and limitations in standardization [24]. In a study done by Reis et al. [23], they reported that there were no statistically significant differences between the time and number of pecking movements between natural teeth and artificial teeth. This suggests that artificial teeth could be an adequate replica for use in endodontic training. For these reasons, artificial teeth were used in this study.

The ability to detect canal orifices and negotiate root canals in minimally invasive endodontic access cavities (MIECs) was reported to be highly dependent on the use of a dental microscope and ultrasonic tips [25]. It has been found that there is no significant difference in canal detection between conservative access and traditional access when the operating microscope was used with the aid of ultrasonic instruments [8,26,27].

Reciprocation motion is reported as a safe and good alternative to traditional continuous rotational motion [15,16]. According to the results of this study, no instrument separation was recorded, which agrees with previous studies that have reported a 0.13%-0.26% lower incidence of fractures when using reciprocal motion compared to systems with continuous rotation motion [28-31].

Coronal dentinal interferences may hinder the instrument's ability to follow the original canal morphology. The reduction in the centering ability of the instrument inside the canals might lead to this deviation from the original canal anatomy, and in turn, this may lead to iatrogenic mishaps [32-34].

Generally, some studies reported a higher incidence of canal transportation [35,36], while others did not [37,38]. Wu et al. reported that canal transportation of greater than 0.3 mm may lead to negative impacts on root canal filling [38].

In this study, canal transportation values were measured for all groups at distances of 3 mm, 6 mm, and 9 mm from the apical foramen. According to the results of this study, a statistically significant difference was found in the conservative access cavity in terms of root canal transportation in the coronal third and middle thirds after root canal preparation (p<0.05), as shown in Figures 2-3, and these results agree with those obtained in the study by Koohnavard et al. [30], who concluded that canal transportation was more in conservative access cavity at all distances from the apical region. Additionally, our results were in accordance with the study by Rover et al., which evaluated the impacts of traditional and conservative access cavities on shaping ability and transportation measures in upper molars [39]. In another study, Lima et al. [31] found that ultraconservative access cavities in mandibular teeth caused more transportation in comparison to traditional cavities. The justification for their results is attributed to the existence of coronal interferences of conservative and ultraconservative access cavities, which cause the deflection of the instruments and lead to the distribution of an uneven force [33,40].

Another study by Krishan et al. concluded that, regardless of the NiTi rotary file system used, there is a negative effect of conservative access cavities on the original canal anatomy in mandibular molars [41].

In contrast, Peng et al. reported no significant difference in terms of canal transportation when using WaveOne Gold in the two different access cavities evaluated [40]. Kadhim et al. reported that traditional access cavities, compared to conservative access cavities, had no statistical difference in transportation and centering ability [42]. The results of our study showed that this was attributed to the presence of more coronal inferences.

The results of the centering ability in this study showed that there was a reduction of centering ability in the conservative access group, as there was a significant difference between TAC and CAC at the coronal third only, which could be attributed to coronal interferences, while there was no significant difference at the middle and apical levels. These results may be due to the heat treatment making the RECIPROC® blue file more flexible, in which it can be bent to follow the natural canal anatomy smoothly in the middle and apical thirds and prepare the canal to the original shape without deviation. This led to enhanced centralization of the rotary file in the root canal. These results disagree with studies conducted by Özyürek et al. and Bayoumi et al. who found no statistical differences between both access cavity designs [43,44].

Additional studies are advised to determine whether minimally invasive access cavities may alter the original morphology of the root canal system in comparison to traditional access cavities. These studies should include larger sample sizes and multiple rotary systems to provide broad insights into the possible differences between these approaches.

## Conclusions

A minimally invasive approach has been adopted as a new perspective in endodontics in recent years. Within the limitations of this in vitro study, the RECIPROC single file performed similarly in traditional and contracted endodontic cavities in terms of centering ability at the middle and apical thirds in the root canals of the maxillary molars. However, for transportation, contracted endodontic cavities negatively affected the original root canal morphology. These results supported the rationale for the revision of the guidelines for the design of endodontic access cavities.

More studies should be conducted on conservative endodontic access before clinical application.
